# Pancreatitis affects gut microbiota via metabolites and inflammatory cytokines: an exploratory two-step Mendelian randomisation study

**DOI:** 10.1007/s00438-024-02125-6

**Published:** 2024-03-16

**Authors:** Yi-Fan Qiu, Jun Ye, Jin-Jin Xie, Xiao-Tong Mao, Yi-Long Liu, Qian Fang, Yang-Yang Qian, Wen-Bin Zou, Yu Cao, Zhuan Liao

**Affiliations:** 1 Department of Gastroenterology, Changhai Hospital, Second Military Medical University/Naval Medical University, 168 Changhai Road, Shanghai, 200433 China; 2 College of Basic Medicine Sciences, Second Military Medical University/Naval Medical University, Shanghai, China

**Keywords:** Cytokines, Gut microbiota, Mendelian randomisation, Pancreatitis, Pancreas–gut axis

## Abstract

Previous studies have observed relationships between pancreatitis and gut microbiota; however, specific changes in gut microbiota abundance and underlying mechanisms in pancreatitis remain unknown. Metabolites are important for gut microbiota to fulfil their biological functions, and changes in the metabolic and immune environments are closely linked to changes in microbiota abundance. We aimed to clarify the mechanisms of gut–pancreas interactions and explore the possible role of metabolites and the immune system. To this end, we conducted two-sample Mendelian randomisation (MR) analysis to evaluate the casual links between four different types of pancreatitis and gut microbiota, metabolites, and inflammatory cytokines. A two-step MR analysis was conducted to further evaluate the probable mediating pathways involving metabolites and inflammatory cytokines in the causal relationship between pancreatitis and gut microbiota. In total, six potential mediators were identified in the causal relationship between pancreatitis and gut microbiota. Nineteen species of gut microbiota and seven inflammatory cytokines were genetically associated with the four types of pancreatitis. Metabolites involved in glucose and amino acid metabolisms were genetically associated with chronic pancreatitis, and those involved in lipid metabolism were genetically associated with acute pancreatitis. Our study identified alterations in the gut microbiota, metabolites, and inflammatory cytokines in pancreatitis at the genetic level and found six potential mediators of the pancreas–gut axis, which may provide insights into the precise diagnosis of pancreatitis and treatment interventions for gut microbiota to prevent the exacerbation of pancreatitis. Future studies could elucidate the mechanism underlying the association between pancreatitis and the gut microbiota.

## Introduction

Pancreatitis is the most prevalent exocrine pancreatic disease that has a major impact on the homeostasis of the digestive system. The destruction of pancreatic structures during the development of pancreatitis causes abnormal pancreatic secretion, which can disrupt intestinal homeostasis and disturb gut microbiota (Akshintala et al. 2019; Capurso et al. 2016). Several clinical studies have reported overall changes in the gut microbiota in patients with pancreatic disease (Tan et al. 2015) and attempted to clarify the specific role of the gut microbiota in pancreatic disease development (Mirji et al. 2022). However, current research on the interaction between gut microbiota and the pancreas is relatively limited. Disturbance of gut microbiota due to pancreatitis and the underlying mechanisms remain to be investigated.

Researchers have preliminarily identified three ways in which gut flora intervenes in the course of pancreatitis. Gut microbiota can migrate directly to the pancreas via the duodenal-pancreatic duct and invade the pancreatic tissue via the mesenteric vein and peri-intestinal lymph nodes (Thomas and Jobin 2020; Diehl et al. 2013), exacerbating chronic pancreatitis (CP). Moreover, disturbed gut microbiota diminishes the production of short-chain fatty acids, leading to inadequate inhibition of the pro-fibrotic function of histone deacetylase and exacerbating CP (Pang and Zhuang 2010; Bombardo et al. 2018). Additionally, short-chain fatty acids and other microbiota productions could influence the inflammatory process by regulating the production of inflammatory cytokines and the differentiation of immune cells (Li et al. 2018; Sun et al. 2018).

Moreover, gut microbiota has been associated with acute pancreatitis (AP). Disturbed gut microbiota causes impaired intestinal barrier and ectopic microbiota, which may be one of the crucial mechanisms by which gut microbiota promote AP (Sonika et al. 2017). Notably, gut microbiota metabolites indirectly influence the course of AP by interacting with immune molecules, such as bifidobacteria, through its metabolite lactate and interfering with the TLR4/MyD88 and NLRP3/Caspase1 pathways to alleviate AP symptoms (Li et al. 2022a). This indicates that metabolites and the immune system may play a pivotal role in the intricate mechanisms through which the gut microbiota intervene in pancreatitis.

The ‘pancreas-gut axis’ concept was first proposed in 2016 (Perry et al. 2016). The authors demonstrated that acetate produced by gut microbiota metabolism could modulate pancreatic β-cell function and increase insulin secretion. A vicious cycle of ‘pancreatitis-gut microbiota disruption-pancreatitis exacerbation’ may exist in patients with pancreatitis. Capurso et al. (2016) reported that small intestinal bacterial overgrowth (SIBO) was present in 36% of CP patients and treating SIBO in pancreatitis patients could improve their exocrine insufficiency symptoms (Bashir et al. 2018). Exploring how pancreatitis alters the abundance of gut microbiota and how these changes, along with the disruption of metabolic homeostasis, influence the progression of pancreatitis is critical. However, most previous studies focussed exclusively on the impact of gut microbiota and associated metabolites on pancreatic diseases. Furthermore, although some observational studies have explored how gut microbiota abundance is altered in pancreatitis patients, these clinical studies often had small sample sizes, high heterogeneity, and inconsistent results across different regions.

Mendelian randomisation (MR), using genetic variants indexing of exposure to establish inter-causality relationships with outcomes (Davey Smith and Hemani 2014; Liu et al. 2022), can overcome the confounding biases inherent in observational studies. Hence, this study aimed to use a two-sample MR analysis based on the publicly available genome-wide association studies (GWAS) database from a large population to illustrate the effect of pancreatitis on the gut microbiota and perform two-step MR analysis to explore the role that metabolites and inflammatory cytokines play in this process. Understanding the specific alteration of gut microbiota abundance due to pancreatic inflammation and the associated mechanisms holds the potential for a breakthrough in alleviating the symptoms of pancreatitis and preventing its exacerbation.

## Materials and methods

### Study design

**Fig. 1:**
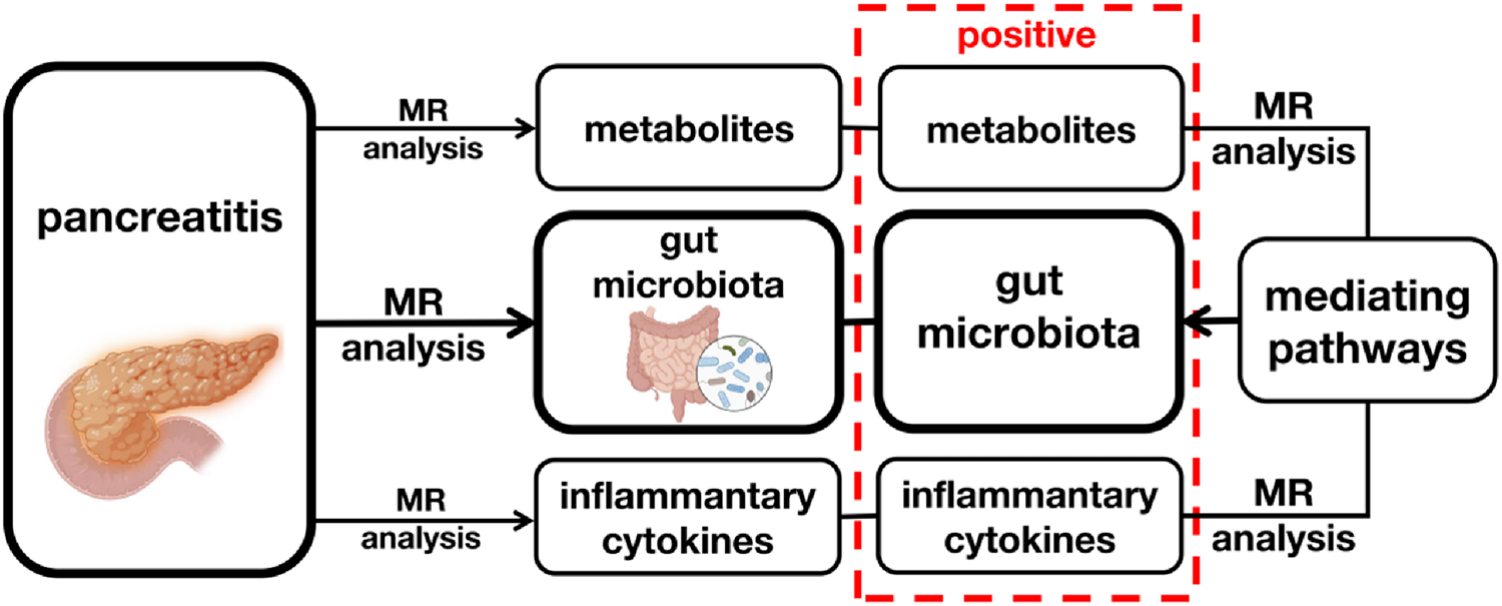
Study design. *MR* Mendelian randomisation

The MR study comprised two phases. In phase 1, we examined the causal effects of all four types of pancreatitis provided by FinnGen GWAS Database (CP, alcohol-induced chronic pancreatitis [AICP], AP, and alcohol-induced acute pancreatitis [AIAP]) on the gut microbiota abundance and metabolites and inflammatory cytokines levels. In phase 2, we assessed the mediating role of metabolites and inflammatory cytokines in the causal relationships between pancreatitis and gut microbiota; in this phase, positive metabolites and inflammatory cytokines in phase 1 were considered as exposures and positive microbiota as outcomes. Generally, we first examined the causal effects of pancreatitis on potential mediators and then assessed the causal effects of mediators on gut microbiota (Fig. 1).

The following criteria should be met in the two-step MR analysis: (1) the exposure should be causally associated with the outcome; (2) the mediator should be causally associated with the outcome independent of the exposure; (3) congruent directions for direct and indirect effects of the exposure on the outcome; and (4) the exposure should be causally associated with the mediator, but not vice versa. In each phase, a simple reverse MR analysis was performed to confirm the absence of reverse causality in the final positive results.

### Data sources for pancreatitis, gut microbiota, inflammatory cytokines, and metabolites

Genetic variables of the gut microbiota were obtained from a GWAS dataset of the International Consortium MiBioGen (Kurilshikov et al. 2021); a large international research programme aimed at studying the impact of human genes on gut microbiota at a genome-wide level. This study included genomic and gut microbiota data from 24 cohorts of 18,340 people from multiple countries and ethnicities in Europe, the USA, the Middle East, and East Asia and is the largest GWAS on gut microbiota to date. Overall, 211 taxa (131 genera, 35 families, 20 orders, 16 classes, and 9 phyla) were included.

GWAS summary statistics for pancreatitis were obtained from the FinnGen Consortium R9 release data (Kurki et al. 2023). The current study incorporated the following phenotypes: AIAP (containing 931 cases and 376,346 controls), AICP (containing 1794 cases and 375,483 controls), AP (containing 6223 cases and 330,903 controls), and CP (containing 3320 cases and 330,903 controls). Patients with these four types of pancreatitis were included according to the diagnostic criteria of the International Classification of Diseases Tenth Edition, and the codes were K85.000 (AP), K85.200 (AIAP), K86.100 (CP), and K86.000 (AICP).

Data on genetic variables for 41 inflammatory cytokines were obtained from a study providing genome variant associations in 8293 Finnish individuals. This study combined the results of the Cardiovascular Risk in Young Finns Study and FINRISK surveys (Ahola-Olli et al. 2017). The metabolite GWAS included 24,925 European participants from 14 cohorts, whose blood metabolites were profiled using a quantitative nuclear magnetic resonance metabolomics platform. Notably, there was no overlap in the population or cohort selection between the exposure and outcome groups (Table 1).

**Table 1:** Data Source and Information of this study

	Ancestry	Sample size (case/control)	Data sources	Web source
Acute pancreatitis	Europe	6223/330,903	FinnGen GWAS Database	https://storage.googleapis.com/finngen-public-data-r9/summary_stats/finngen_R9_K11_ACUTPANC.gz
Chronic pancreatitis	Europe	3320/330,903	FinnGen GWAS Database	https://storage.googleapis.com/finngen-public-data-r9/summary_stats/finngen_R9_K11_CHRONPANC.gz
Alcohol-induced acute pancreatitis	Europe	931/376,346	FinnGen GWAS Database	https://storage.googleapis.com/finngen-public-data-r9/summary_stats/finngen_R9_ALCOPANCACU.gz
Alcohol-induced chronic pancreatitis	Europe	1794/375,483	FinnGen GWAS Database	https://storage.googleapis.com/finngen-public-data-r9/summary_stats/finngen_R9_ALCOPANCCHRON.gz
Gut microbiota	Mixed (94% Europe)	18,340	Alexander Kurilshikov et al.	https://mibiogen.gcc.rug.nl/menu/main/home
Metabolites	Europe	24,925	Johannes Kettunen et al.	https://gwas.mrcieu.ac.uk
Inflammatory cytokines	Europe	8293	AriV. Ahola-Olli et al.	https://data.bris.ac.uk/data/dataset/3g3i5smgghp0s2uvm1doflkx9x

### Selection of instrumental variables

The following criteria were used to select the optimal instrumental variables (IVs) to improve the authenticity and accuracy of the study conclusions. (1) When we selected pancreatitis as the exposure, we used a genome-wide statistical significance threshold (*P* < 5 × 10^−8^) to select IVs. When we chose inflammatory cytokines as the exposure, we adjusted the threshold downwards to a locus-wide significance level (*P* < 5 × 10^−6^) if a few relevant single-nucleotide polymorphisms (SNPs) were identified. When the gut microbiome data were defined as the exposure, the threshold of *P* values was relaxed to 1 × 10^–5^ to ensure that a suitable number of SNPs were included in the analysis. (2) Because the presence of strong linkage disequilibrium (LD) might result in bias, we ensured that there was no LD among the selected IVs. Data from the 1000 Genomes Project European samples were used as the reference panel to calculate the LD between the SNPs, and the SNPs reaching the threshold (*r*^2^ < 0.01, kb = 10,000) were retained for subsequent analysis. (3) The *F*-statistics for the selected IVs reached a threshold of > 10, ensuring that the causal estimations had no weak instrument bias. Sufficient SNPs were used in our analysis, and their numbers are shown in the figures.

### MR analysis

**Fig. 2:**
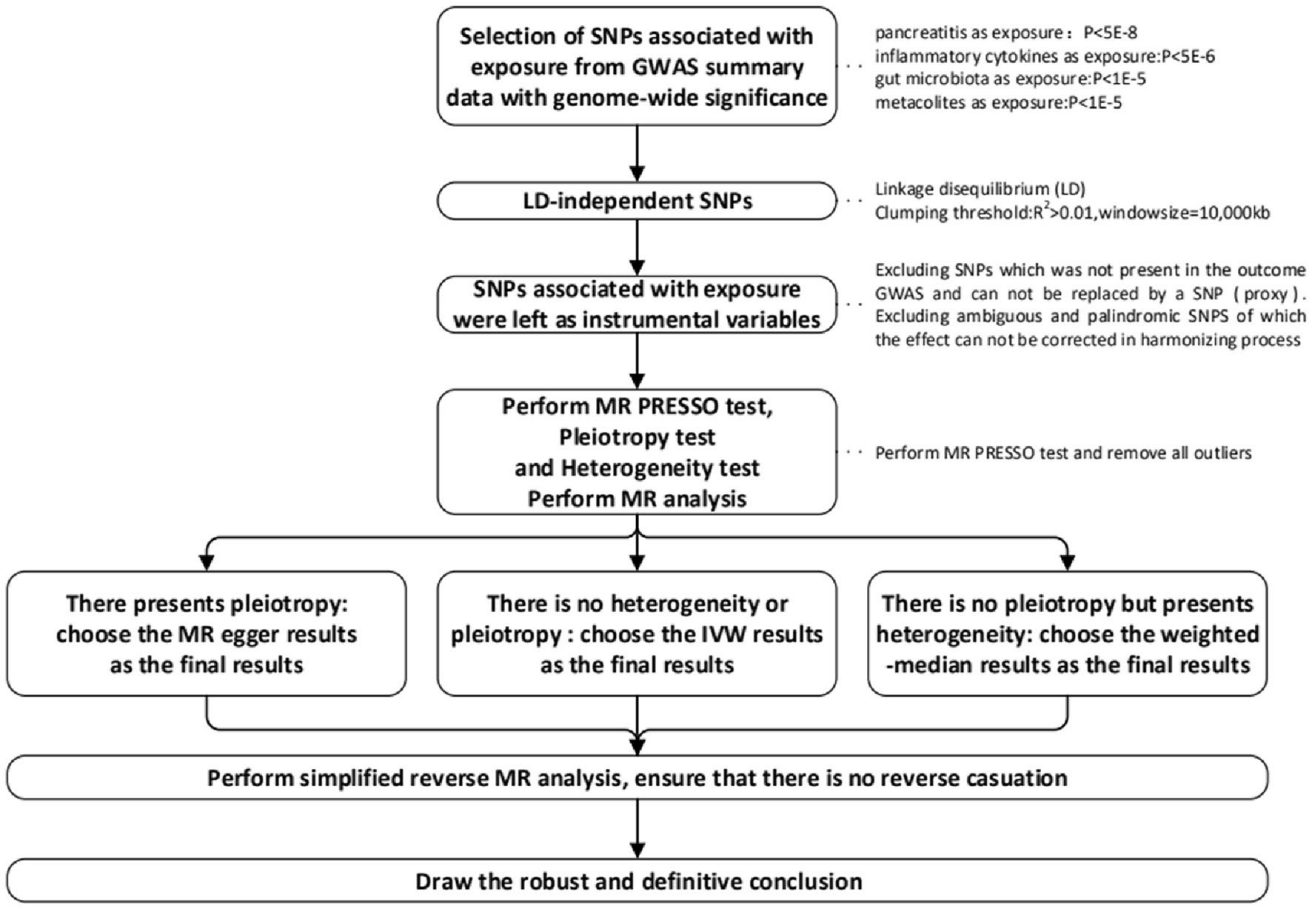
Process of Mendelian randomization

This study used a two-step MR method. The phylum, family, and genus levels were included in the analysis. Metabolites and inflammatory cytokines were considered mediators of the causal effects of pancreatitis on gut microbiota. First, we performed an MR pleiotropy residual sum and outlier (MR-PRESSO) test to remove all outlier SNPs (Verbanck et al. 2018). Second, pleiotropy and heterogeneity tests were conducted to ensure the robustness of the results. Cochran’s *Q*- and *Q*-derived *P* values were calculated to assess heterogeneity (Greco et al. 2015), and MR-Egger intercept *P* values were calculated to assess pleiotropy (Bowden et al. 2015). The MR-Egger results were accepted when the genetic variants had pleiotropic effects. Finally, five popular MR methods were used to analyse features containing multiple IVs: the inverse-variance weighted (IVW) test, weighted mode, MR-Egger regression, weighted median estimator, and simple mode. In our analyses, there were no cases in which the number of SNPs was less than three; hence, all the above methods were available (Fig. 2). In some cases, IVW was considered a method with relatively high statistical validity; therefore, our analysis was based primarily on IVW results (Greco et al. 2015), with the other four methods acting as complementarity methods. We used the coefficient method as our main method to estimate the mediation effect (VanderWeele 2016) and the delta method to calculate the value of mediation proportion (Burgess et al. 2015).

### Ethics

This study used publicly available data from specific human experimentation studies approved by the Ethical Standards Committee. Accordingly, ethical approval was not required for this study.

### Statistical analysis

All analyses were performed using the packages TwoSampleMR (version 0.5.6) and MR-PRESSO (version 1.0) in R (version 4.2.2) Studio. Indirect effect of the intermediates was estimated using the website of the RMediation (https://amplab.shinyapps.io/MEDCI/), and the package RMediation (version 1.2.2) could also function similarly. The false discovery rate q value was calculated using the Benjamini–Hochberg method to test multiple hypotheses.

## Results

### Causal effects of pancreatitis on gut microbiota

#### Causal effects of CP on gut microbiota

At the phylum level, CP occurrence was genetically associated with a low abundance of *Lentisphaerae* (*β* = − 1.013, standard error (SE) = 0.051, *P* value = 0.046). At the genus level, CP occurrence was genetically associated with a low abundance of *Ruminococcus torques* (*β* = − 0.071, SE = 0.023, *P* value = 0.0016). Contrastingly, CP occurrence was genetically associated with a high abundance of *Eubacterium brachy* (*β* = 0.152, SE = 0.069, *P* value = 0.028) and *Lachnospiraceae UCG001* (*β* = 0.065, SE = 0.030, *P* value = 0.030).

#### Causal effects of AICP on gut microbiota

At the genus level, AICP occurrence was genetically associated with a low abundance of *Candidatus soleaferrea* (*β* = − 0.079, SE = 0.029, *P* value = 0.007) and *Eubacterium fissicatena* (*β* = − 0.152, SE = 0.059, *P* value = 0.009). Conversely, AICP occurrence was genetically associated with a high abundance of *Eubacterium brachy* (*β* = 0.152, SE = 0.069, *P* value = 0.029) and *Lachnospiraceae UCG001* (*β* = 0.065, SE = 0.030, *P* value = 0.030). Furthermore, AICP occurrence was genetically associated with a high abundance of *Eubacterium brachy* (*β* = 0.121, SE = 0.047, *P* value = 0.011), *Lachnospiraceae UCG010* (*β* = 0.048, SE = 0.020, *P* value = 0.016), *Erysipelotrichaceae UCG003* (*β* = 0.047, SE = 0.021, *P* value = 0.024), *Lachnospiraceae UCG004* (*β* = 0.041, SE = 0.019, *P* value = 0.029), *Eggerthella* (*β* = 0.067, SE = 0.032, *P* value = 0.035), *Eggerthella* (*β* = 0.067, SE = 0.032, *P* value = 0.035), and *Anaerostipes* (*β* = 0.034, SE = 0.018, *P* value = 0.049).

#### Causal effects of AP on gut microbiota

At the family level, AP occurrence was genetically associated with a high abundance of *Bacteroidaceae* (*β* = 0.060, SE = 0.030, *P* value = 0.049). At the phylum level, AP occurrence was genetically associated with a high abundance of *Proteobacteria* (*β* = 0.073, SE = 0.027, *P* value = 0.007). At the genus level, AP occurrence was genetically associated with a low abundance of *Candidatus soleaferrea* (*β* = − 0.100, SE = 00.047, *P* value = 0.033), and a high abundance of *Odoribacter* (*β* = 0.069, SE = 0.031, *P* value = 0.024), *Butyricicoccus* (*β* = 0.060, SE = 0.028, *P* value = 0.034), and *Bacteroides* (*β* = 0.060, SE = 0.030, *P* value = 0.049).

#### Causal effects of AIAP on gut microbiota

**Fig. 3:**
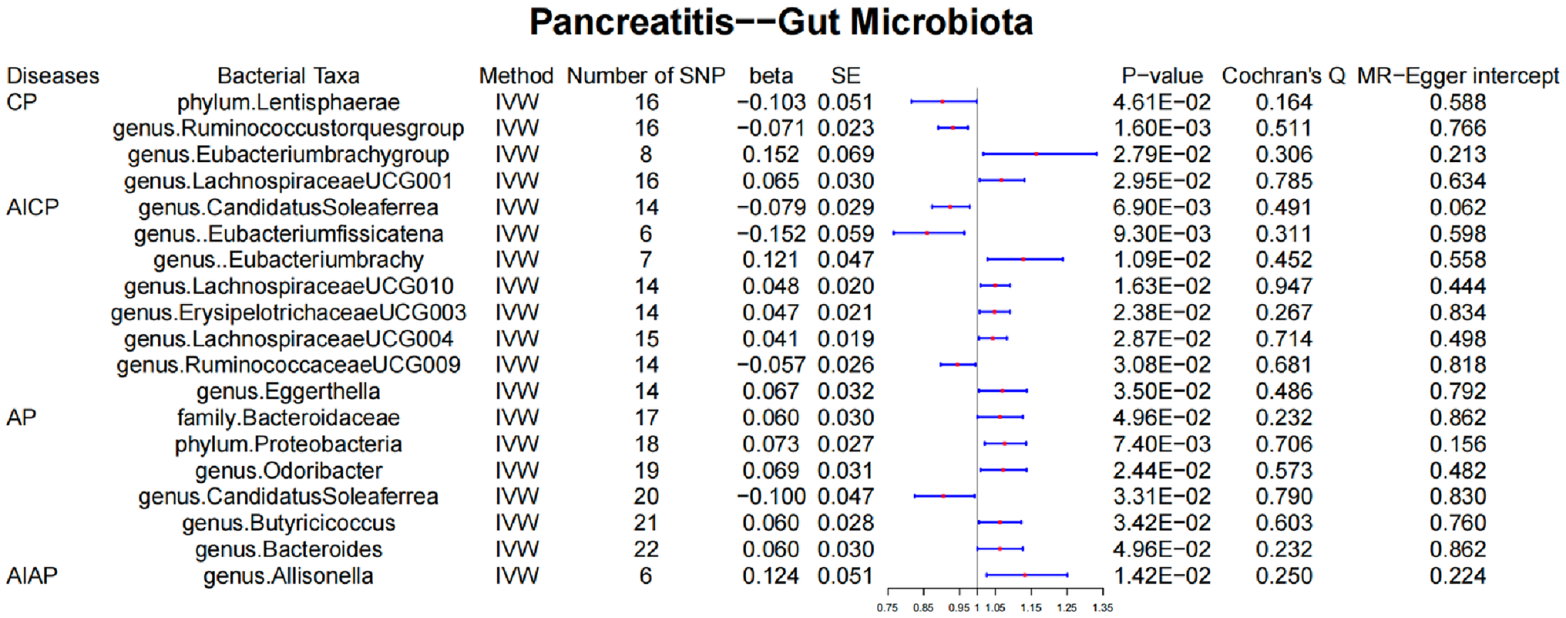
Causal effects of pancreatitis on gut microbiota. *CP* chronic pancreatitis, *AICP* alcohol-induced chronic pancreatitis, *AP* acute pancreatitis, *AIAP* alcohol-induced acute pancreatitis, *SE* standard error

At the family level, AIAP occurrence was genetically associated with a high abundance of *Allisonella* (*β* = 0.124, SE = 0.051, *P* value = 0.0142). The Cochran’s *Q*-deprived and MR-Egger intercept *P* values showed no pleiotropy or heterogeneity in the causal effects of pancreatitis on gut microbiota. Simple reverse MR was performed, showing no reverse causality of the gut microbiota on pancreatitis. Some SNPs may overlap owing to the genus, and Butyricicoccus was a child taxon of the family Bacteroidaceae, which was revealed by their same *β*, SE, and *P* values (Fig. 3).

### Causal effects of pancreatitis on metabolites

#### Causal effects of CP on metabolites

CP occurrence was genetically associated with a low serum level of citrate (*β* = − 0.032, SE = 0.014, *P* value = 0.025).

#### Causal effects of AICP on metabolites

AICP occurrence was genetically associated with a high serum level of pyruvate (*β* = 0.025, SE = 0.012, *P* value = 0.028). Contrastingly, AICP occurrence was genetically associated with a low serum level of valine (*β* = − 0.025, SE = 0.011, *P* value = 0.020), tyrosine (*β* = − 0.024, SE = 0.011, *P* value = 0.031), and leucine (*β* = − 0.023, SE = 0.011, *P* value = 0.031).

#### Causal effects of AP on metabolites

AP occurrence was genetically associated with a low serum level of apolipoprotein A-I (*β* = − 0.039, SE = 0.019, *P* value = 0.044); omega-3 fatty acids (*β* = − 0.062, SE = 0.025, *P* value = 0.015); omega-6 fatty acids (*β* = − 0.049, SE = 0.024, *P* value = 0.036); omega-7, omega-9 and saturated fatty acids (*β* = − 0.055, SE = 0.023, *P* value = 0.016); free cholesterol (*β* = − 0.078, SE = 0.027, *P* value = 0.036); free cholesterol (*β* = − 0.078, SE = 0.027, *P* value = 0.036); free cholesterol in intermediate-density lipoprotein (IDL) (*β* = − 0.053, SE = 0.027, *P* value = 0.046); cholesterol esters in large high-density lipoprotein (HDL) (*β* = − 0.039, SE = 0.019, *P* value = 0.040); mono-unsaturated fatty acids (*β* = − 0.055, SE = 0.023, *P* value = 0.015); total cholesterol (*β* = − 0.054, SE = 0.024, *P* value = 0.025); total fatty acids (*β* = − 0.060, SE = 0.024, *P* value = 0.011); total cholesterol in very large HDL (*β* = − 0.045, SE = 0.018, *P* value = 0.015); cholesterol esters in very large HDL (*β* = − 0.056, SE = 0.019, *P* value = 0.003); free cholesterol in very large HDL (*β* = − 0.051, SE = 0.018, *P* value = 0.006); total lipids in very large HDL (*β* = − 0.053, SE = 0.019, *P* value = 0.005); phospholipids in very large HDL (*β* = − 0.049, SE = 0.019, *P* value = 0.009); and triglycerides in very large HDL (*β* = − 0.049, SE = 0.019, *P* value = 0.009).

#### Causal effects of AIAP on metabolites

**Fig. 4:**
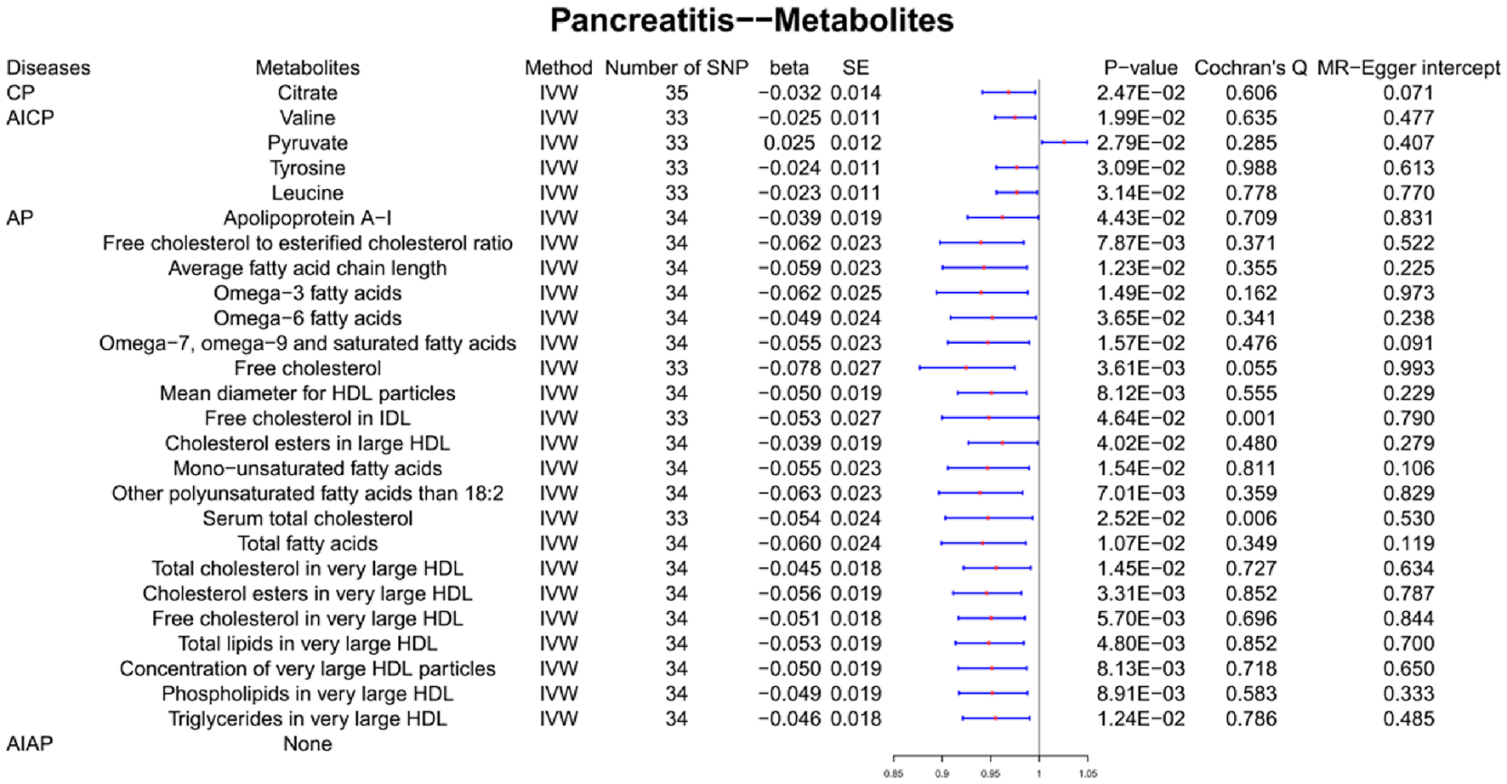
Causal effects of pancreatitis on metabolites. *CP* chronic pancreatitis, *AICP* alcohol-induced chronic pancreatitis, *AP* acute pancreatitis, *AIAP* alcohol-induced acute pancreatitis, *SE* standard error

There was no causal effect of AIAP occurrence on metabolites. The MR-PRESSO test excluded one SNP in the analysis of the causal effects of AP on free cholesterol, free cholesterol in IDL, and total cholesterol, and the resulting *P* value remained < 0.05. There was heterogeneity in the causal effects of AP on free cholesterol in the IDL and total cholesterol, whereas the *β* values of the five MR methods were all negative; therefore, these two metabolites remained in the final results (Fig. 4).

### Causal effects of pancreatitis on inflammatory cytokines

#### Causal effects of CP on inflammatory cytokines

CP occurrence was genetically associated with a high serum level of IL-4 (*β* = 0.048, SE = 0.024, *P* value = 0.048), SCGF-*β* (*β* = 0.077, SE = 0.036, *P* value = 0.034), IL-12-P70 (*β* = 0.048, SE = 0.024, *P* value = 0.043), and IL-10 (*β* = 0.049, SE = 0.025, *P* value = 0.045).

#### Causal effects of AICP on inflammatory cytokines

There was no causal effect of AICP occurrence on inflammatory cytokines.

#### Causal effects of AP on inflammatory cytokines

AP occurrence was genetically associated with high serum levels of IL-4 (*β* = 0.083, SE = 0.036, *P* value = 0.022) and IL-6 (*β* = 0.071, SE = 0.035, *P* value = 0.042).

#### Causal effects of AIAP on inflammatory cytokines

**Fig. 5:**
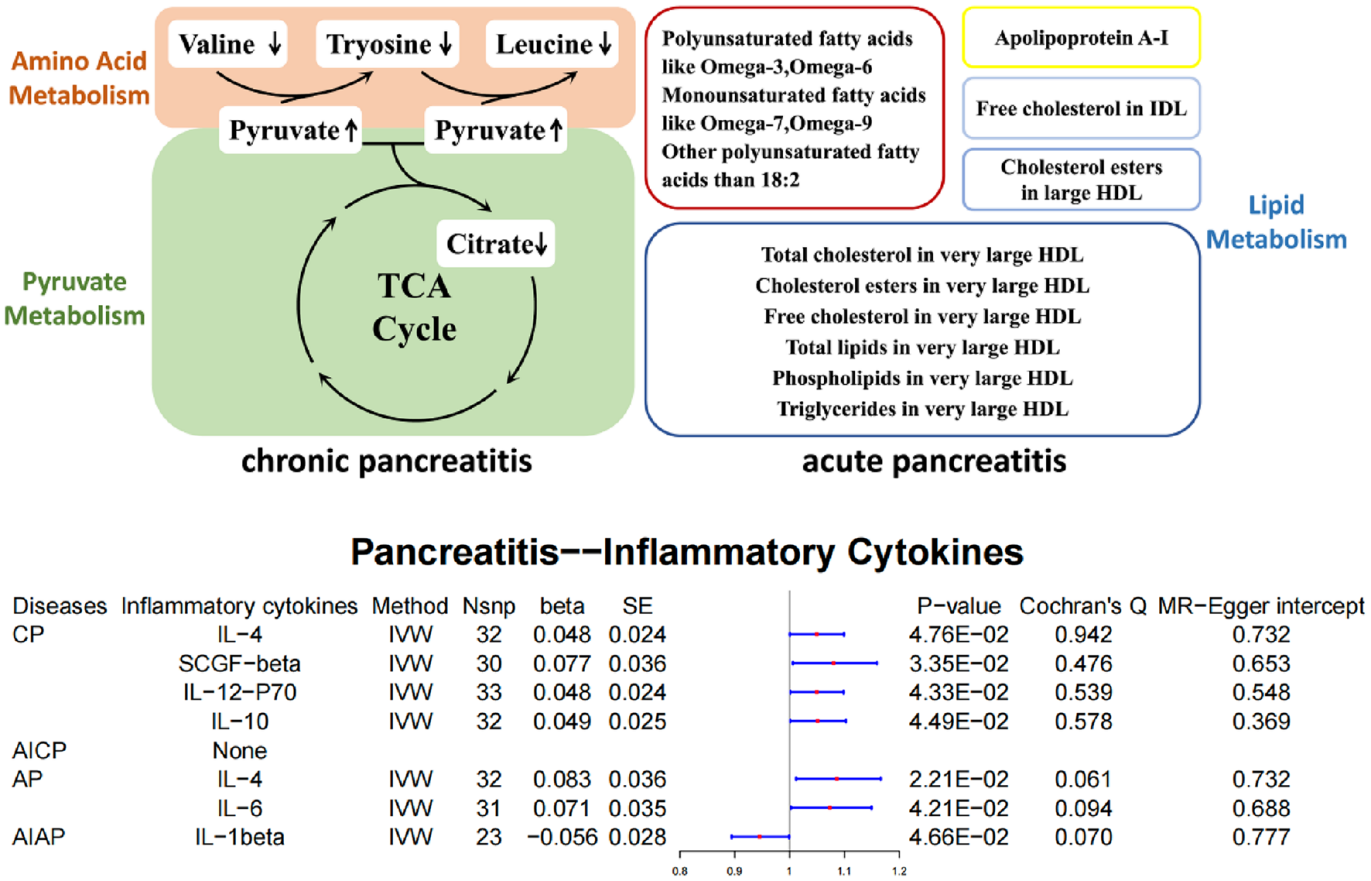
Causal effects of pancreatitis on inflammatory cytokines. *CP* chronic pancreatitis, *AICP* alcohol-induced chronic pancreatitis, *AP* acute pancreatitis, *AIAP* alcohol-induced acute pancreatitis, *SE* standard error

AIAP occurrence was genetically associated with a low serum level of IL-1*β* (*β* = − 0.056, SE = 0.028, *P* value = 0.047) (Fig. 5).

### Potential intermediates in causal effects of pancreatitis on gut microbiota

After a series of processes for exploring the exposure–pancreatitis–outcome gut microbiota causal pathways, we applied a two-step MR to assess the role of inflammatory cytokines and metabolites in mediating the causal effects of pancreatitis on the gut microbiota (Tables 2, 3).

**Table 2:** Inflammatory cytokines as intermediates in causal effects of pancreatitis on gut microbiota

Exposure	*β*_e-i_	Intermediate	*β*_i-o_	Outcome	*β*_e-o_	*β*	SE	Intermediate ratio (%)
AICP	0.025	Pyruvate	0.286	genus.*Eggerthella*	0.067	0.007	0.013	10.4
AP	− 0.051	Free cholesterol in very large HDL	0.097	genus.*Candidatus soleaferrea*	− 0.100	− 0.005	0.003	0.5

*AICP* alcohol-induced chronic pancreatitis, *AIAP* alcohol-induced acute pancreatitis, *SE* standard error

**Table 3:** Metabolites as intermediates in causal effects of pancreatitis on gut microbiota

Exposure	*β*_e-i_	Intermediate	*β*_i-o_	Outcome	*β*_e-o_	*β*	SE	Intermediate ratio (%)
CP	0.077	SCGF-*β*	− 0.332	phylum.*Lentisphaerae*	− 0.103	− 0.026	0.013	25.2
	0.048	IL-12-P70	0.028	genus.*Eubacterium brachy*	0.152	0.001	0.001	0.6
	0.048	IL-4	0.304	genus.*Lachnospiraceae UCG001*	0.065	0.032	0.022	49.2
AP	0.071	IL-6	0.200	phylum.*Proteobacteria*	0.073	0.014	0.009	19.2

*CP* chronic pancreatitis, *AP* acute pancreatitis, *SCGF* stem cell growth factor, *SE* standard error

#### Inflammatory cytokines as intermediates in causal effects of pancreatitis on gut microbiota

**Fig. 6:**
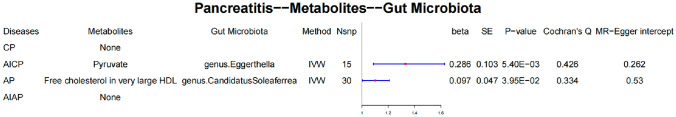
Metabolites as intermediates in causal effects of pancreatitis on gut microbiota. *CP* chronic pancreatitis, *AICP* alcohol-induced chronic pancreatitis, *AP* acute pancreatitis, *AIAP* alcohol-induced acute pancreatitis, *SE* standard error

After the MR-egger adjustment, the analysis showed that IL-4 (*β* = 0.032, SE = 0.022) accounted for 49.2% of the total effect of CP on *Lachnospiraceae*; SCGF-*β* (*β* = − 0.026, SE = 0.013) for 25.2% of the total effect of CP on *Lentisphaerae*, and IL-12-P70 (*β* = 0.001, SE = 0.001) for 0.6% of the total effect of CP on *Eubacterium brachy*. Moreover, IL-6 (*β* = 0.014, SE = 0.009) accounted for 19.2% of the total effect of AP on *Proteobacteria* (Fig. 6).

#### Metabolites as intermediates in causal effects of pancreatitis on gut microbiota

**Fig. 7:**
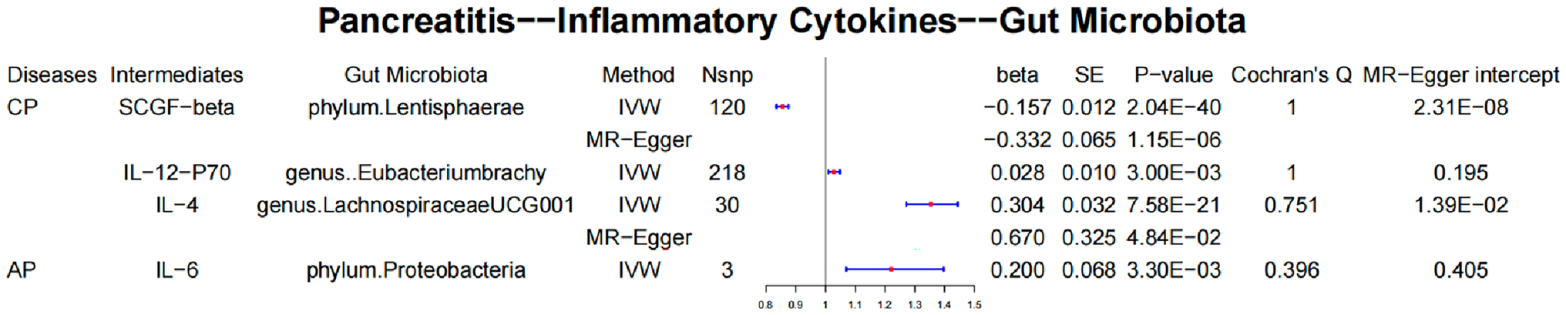
Inflammatory cytokines as intermediates in causal effects of pancreatitis on gut microbiota. *CP* chronic pancreatitis, *AICP* alcohol-induced chronic pancreatitis, *AP* acute pancreatitis, *AIAP* alcohol-induced acute pancreatitis, *SE* standard error

The analysis showed that pyruvate (*β* = 0.007, SE = 0.013) accounted for 10.4% of the total effect of AICP on *Eggerthella* and free cholesterol in very large HDL (*β* = − 0.005, SE = 0.003) for 0.5% of the total effect of CP on *Candidatus soleaferrea* (Table 3, Fig. 7).

## Discussion

This study focussed on the causal effects of pancreatitis on the gut microbiota, analysing the independent causal effects of pancreatitis on gut microbiota abundance, serum metabolite levels, and inflammatory cytokine levels. Furthermore, this study addressed whether metabolites and inflammatory cytokines play mediating roles in promoting gut microbiota disruption.

**Fig. 8:**
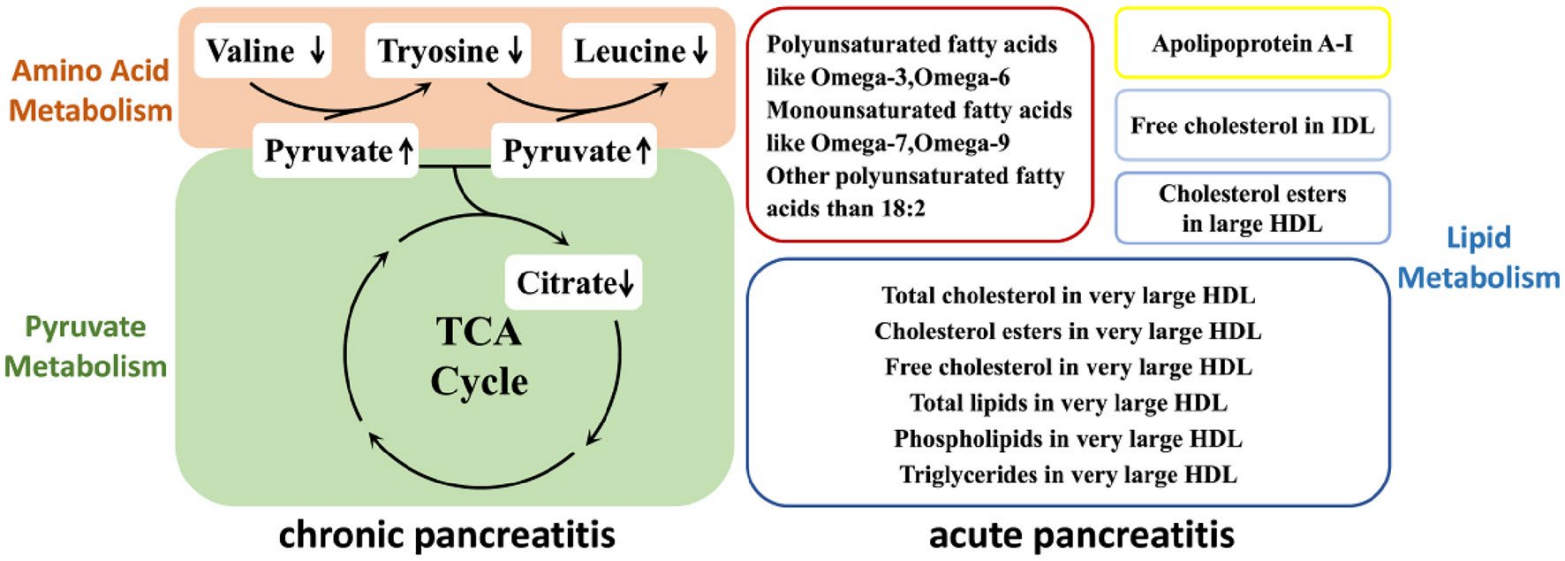
Causal effects of pancreatitis on metabolites. *CP* chronic pancreatitis, *AICP* alcohol-induced chronic pancreatitis, *AP* acute pancreatitis, *AIAP* alcohol-induced acute pancreatitis

Regarding metabolites, our study demonstrated that CP was genetically associated with a low serum level of citrate and AICP was genetically associated with a high serum level of pyruvate and low serum levels of valine, tyrosine, and leucine. Pyruvate is the key molecule that links glycolysis to the tricarboxylic acid (TCA) cycle (Fig. 8). In addition to its participation in the TCA cycle, pyruvate is a vital component of the phenylalanine metabolic pathway. Valine and leucine can be broken down into pyruvate for subsequent reactions, and pyruvate can be used as a reactant in amino acid conversion reactions. Another MR analysis has reported a strong association between plasma alanine and glutamic acid and the abundance of gut *Proteobacteria*, which complements our study and further validates the complex relationship between pancreatitis, amino acid metabolism, and gut microbiota disorders (Liu et al. 2022). Additionally, clinical studies with small sample sizes have confirmed reduced serum citrate and tyrosine concentrations in CP patients (Xu et al. 2023), and some basic studies have also shown that activated pancreatic stellate cells promote glycolysis and upregulate pyruvate production in the pancreatic tissues of CP patients (Tao et al. 2021). However, how glycolysis, the TCA cycle, and amino acid metabolism in pancreatic acinar cells influence each other during the development of pancreatitis remains unclear.

The lipids genetically associated with AP can be grouped into three main categories: unsaturated fatty acids, cholesterol, and HDL. Unsaturated fatty acids may be beneficial in the development and prognosis of AP. Alhan et al. (2006) found that feeding omega-3 fatty acids reduced mortality in rats that developed acute necrotising pancreatitis and lowered IL-6 levels, corroborating with our analysis results on the potential causal relationship between AP and inflammatory cytokines. IL-6 may reduce the expression of Apo A1, which may lead to a decrease in circulating HDL-C levels (Navarro et al. 2005). Hypertriglyceridaemia is a complication of AP; however, our study showed that low levels of triglycerides in very large HDL were genetically associated with AP, which could be explained by the low levels of very large HDL, although the specific mechanisms involved remain unclear.

**Fig. 9:**
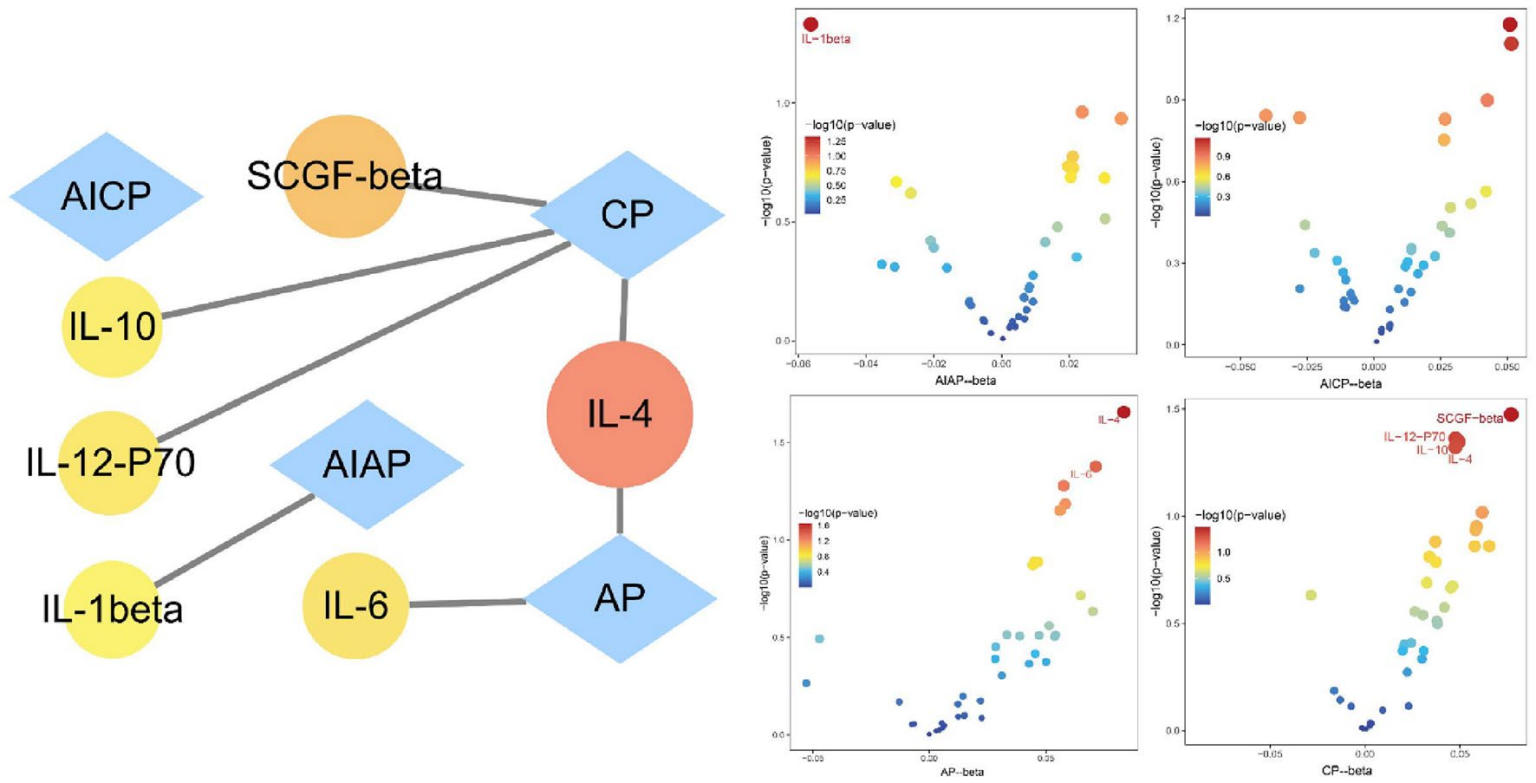
Causal effects of pancreatitis on inflammatory cytokines. *CP* chronic pancreatitis, *AICP* alcohol-induced chronic pancreatitis, *AP* acute pancreatitis, *AIAP* alcohol-induced acute pancreatitis, *SCGF* stem cell growth factor

In addition to the metabolites, elevated levels of IL-4, IL-12-P70, IL-10, and SCGF-*β* were genetically associated with CP (Fig. 9). IL-4 promotes alternative activation of macrophages into M2 cells, and activated M2 cells are associated with fibrosis (Xue et al. 2015). High levels of IL-10 have been detected in CP patients (Tanţău et al. 2021), indicating that when CP occurs, IL-10 secretion may be upregulated to reduce tissue damage. IL-12 stimulates the production of IFN-γ and TNF-α and reduces IL-4-mediated suppression of IFN-γ. IL-6 is released by various immune cells in response to tissue damage and can be a marker for predicting the severity and prognosis of AP (Li et al. 2022b). A low level of IL-1*β* was genetically associated with AIAP, which may be because TLR2 deficiency in a mouse model of cerulein-induced AP decreased the expression of IL-1*β* (Li et al. 2023).

**Fig. 10:**
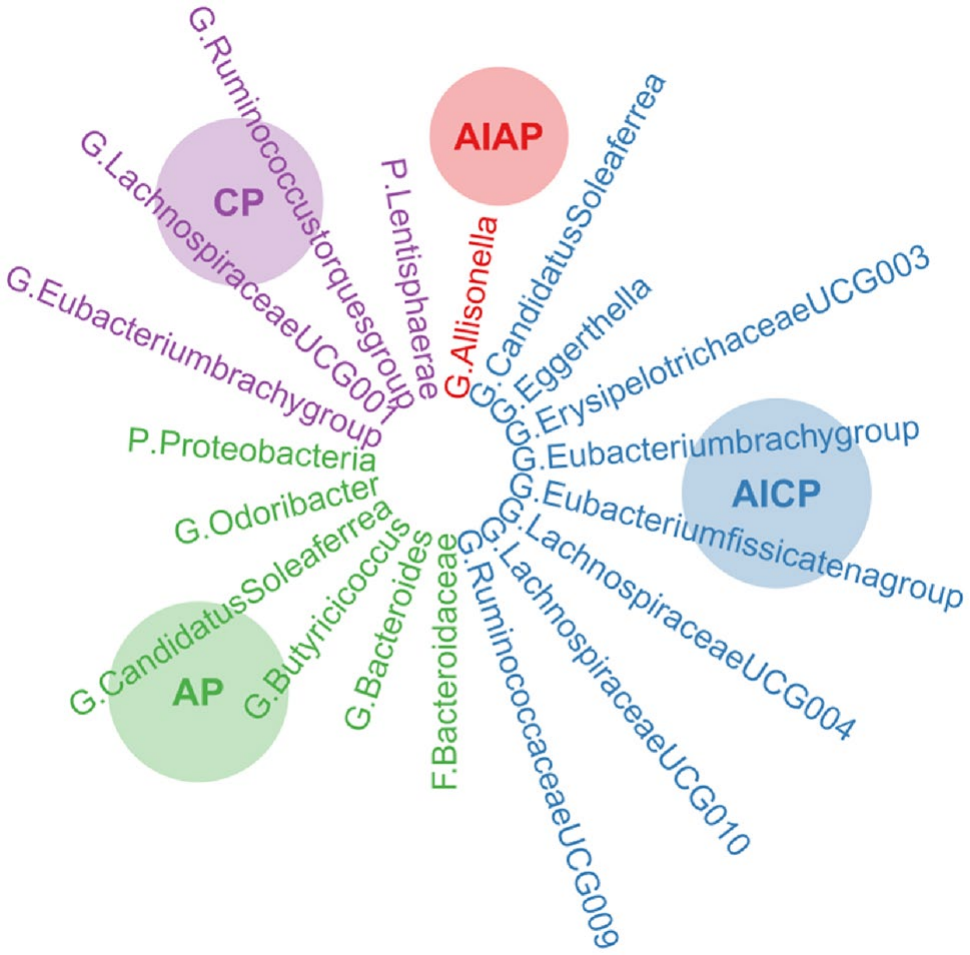
Causal effects of pancreatitis on gut microbiota. *CP* chronic pancreatitis, *AICP* alcohol-induced chronic pancreatitis, *AP* acute pancreatitis, *AIAP* alcohol-induced acute pancreatitis

The four types of pancreatitis are associated with different gut microbiota disorders (Fig. 10). Our study showed that CP was genetically associated with a decreased abundance of the phylum *Lentisphaerae* and genus *Ruminococcus torques* group. A decreased abundance of *Lentisphaerae* has been observed in patients with autoimmune hepatitis (Lou et al. 2020). *Lentisphaerae* may be associated with digestive system diseases; however, the exact mechanism underlying its interaction with CP requires further investigation. *Ruminococcus torques* belongs to the phylum *Mycobacterium* and has been reported to be associated with Crohn’s and non-alcoholic fatty liver disease (Zhang et al. 2023); however, it has no clear relationship with CP. Notably, CP was genetically associated with high abundance of *Eubacterium brachy* and *Lachnospiraceae UCG001*. *Eubacterium* produces short-chain fatty acids including butyric acid and has been reported to have significant differences between CP patients with and without pancreatic exocrine insufficiency (Jandhyala et al. 2017). *Lachnospiraceae* is another gut microbiota that produces butyric acid and is reported to dominate the gut microbiota in CP patients with mild exocrine pancreatic insufficiency (Maev et al. 2023)*.*

AICP was genetically associated with a decreased abundance of the genus *Candidatus soleaferrea*, *Eubacterium fissicatena*, and *Ruminococcaceae UCG009. Candidatus soleaferrea* was reported in a study on mental illnesses, including delirium, schizophrenia, and autism (Yu et al. 2023; Kowalski et al. 2023); therefore, its relationship with AICP might be associated with the effects of alcohol on the nervous system. The increased abundances of *Erysipelotrichaceae* and *Eggerthella* were genetically associated with AICP, which has also been observed in sleep-deprived mice (Cammann et al. 2023). Psychotropic drugs have been shown to reduce the abundance of *Erysipelotrichaceae* (Ait Chait et al. 2023)*.* Therefore, the additional effect of AICP on gut microbiota disorders may be twofold: alteration of the raw material of gut microbiota metabolism by long-term ethanol intake and further effect of alcohol on the nervous system through the brain–gut axis.

This study demonstrated that the abundances of *Bacteroides*, *Proteobacteria*, *Odoribacter*, and *Butyricicoccus* were genetically increased in AP. The small intestine and colon of AP rats had a ‘core microbiota’ composed of bacteria belonging to *Bacteroidetes* and *Proteobacteria* (Tao et al. 2019)*. Odoribacter* is a gut microbiota associated with glucose-lipid metabolism. Huber-Ruano et al. (2022) reported the potential positive effects of *Odoribacter* on insulin sensitivity in individuals with glucose tolerance and obesity. However, there are no current studies on the relationship between AP and *Butyricicoccus*, although researchers have considered *Butyricicoccus* as a potentially exploitable probiotic given its ability to produce butyric acid and its tolerance to the gastrointestinal environment (Geirnaert et al. 2014). Disturbances in the gut microbiota of AP patients may be caused by several factors. Oxidative stress, which may be caused by acute inflammation, may lead to changes in the environment in which the gut microbiota grows and nutrients are available. Damage to the intestinal barrier may allow some of the gut microbiota to expand their growth and colonisation, increasing their detection rate. Additionally, a disease-induced decrease in the abundance of some microbiota may indirectly lead to an increase in the abundance of other microbiota.

The impact of metabolites and inflammatory cytokines on the gut microbiota is substantial. Our study demonstrated that pyruvate may act as a mediator between the causal effects of AICP and *Eggerthella*. Pyruvate is one of the downstream products of alcohol metabolism, and another study on the therapeutic effects of quercetin showed that its use in an antibiotic-treated mouse model unregulated both pyruvate metabolism and the abundance of *Eggerthella* (Mi et al. 2022). Free cholesterol in very large HDL may play a role as a mediator between the causal effects of AP on *Candidatus soleaferrea*; however, no current study has reported the underlying mechanism. SCGF-*β* may play a role as a mediator between the causal effects of CP on *Lentisphaerae*; IL-12 may play a role as a mediator between the causal effects of CP on *Eubacterium*, and IL-4 may play a role as a mediator between the causal effects of CP on *Lachnospiraceae UCG001.* Further, IL-6 may mediate the causal effects of AP on *Proteobacteria*. Butyric acid inhibits IL-12 production, which may explain the mediating effect observed in this study (Singh et al. 2022). The specific role of inflammatory cytokines in the association between diseases and gut microbiota needs to be explored in future research.

Notably, certain estimations varied from logical expectations. We observed that pancreatitis may lead to an increase in the abundance of certain probiotics. There are two possible reasons for this observation. First, the disease prompts this fraction of probiotics to proliferate and fulfil their protective roles. Second, the disease caused a decrease in the abundance of some gut bacteria, indirectly increasing the abundance of this probiotic fraction. Additionally, although there are few reports showing that *Candidatus soleaferrea* is able to metabolise lipids directly, free cholesterol in very large HDL appeared to play a mediating role in our analysis; we speculate that there may be other complex indirect roles.

This study had some limitations. First, most of the enrolled patients were European; therefore, the causal relationship between pancreatitis and gut microbiota in other populations remains unknown. Second, our findings only reported the alteration of gut microbiota in pancreatitis patients and identified several metabolites and inflammatory factors with possible mediating effects; however, the underlying mechanisms warrant further investigation. Finally, GWAS data on metabolites and inflammatory cytokines do not fully represent organ-local conditions; therefore, some changes and pathways that are uniquely significant in the pancreas may be overlooked.

To the best of our knowledge, this is the first study to implement MR analysis to address the causal relationship between gut microbiota, metabolites, inflammatory cytokines, and pancreatitis. This analysis compensates for the disadvantage of using the small sample size in previous clinical studies and adds to the doctrine of pancreas–gut axis at the level of gut microbiota. This research aimed to elucidate the alterations in gut microbiota abundance across four types of pancreatitis patients and investigate the influence of metabolites and inflammatory cytokines in this process. Our findings enhance the understanding of the ‘pancreas-gut’ axis mechanism and offer a foundation for further examination of the role of gut microbiota. Additionally, this work suggests novel avenues for developing targeted therapies for pancreatitis by examining the impact of metabolites and inflammatory cytokines. Given that gut microbiota abundance varies among different types of pancreatitis patients, assessing microbiota abundance could not only shed light on the pathogenesis of pancreatitis but also potentially form a basis for customized microbiota supplementation strategies in treatment.

In conclusion, 19 species of gut microbiota and 7 inflammatory cytokines were genetically associated with the 4 types of pancreatitis. Metabolites involved in glucose and amino acid metabolisms were genetically associated with CP, and those involved in lipid metabolism were genetically associated with AP. Six potential mediators were identified in the causal effects of pancreatitis on the gut microbiota. Accordingly, avoiding further exacerbation of pancreatitis by correcting disturbances in the gut microbiota can be part of a new treatment plan for pancreatitis. The mechanisms underlying the association between the gut microbiota, metabolites, inflammatory cytokines, and pancreatitis require further studies.

## Data Availability

All data are publicly available.
